# Mars Science Laboratory Observations of Chloride Salts in Gale Crater, Mars

**DOI:** 10.1029/2019GL082764

**Published:** 2019-10-07

**Authors:** N. H. Thomas, B. L. Ehlmann, P.‐Y. Meslin, W. Rapin, D. E. Anderson, F. Rivera‐Hernández, O. Forni, S. Schröder, A. Cousin, N. Mangold, R. Gellert, O. Gasnault, R. C. Wiens

**Affiliations:** ^1^ Division of Geological and Planetary Sciences California Institute of Technology Pasadena CA USA; ^2^ Jet Propulsion Laboratory California Institute of Technology Pasadena CA USA; ^3^ Institut de Recherche en Astrophysique et Planétologie, Université de Toulouse, CNRS, UPS, CNES Toulouse France; ^4^ Department of Earth Sciences Dartmouth College Hanover NH USA; ^5^ German Aerospace Center (DLR) Berlin Germany; ^6^ Laboratoire de Planétologie et Géodynamique, UMR6112, CNRS, Université de Nantes Nantes France; ^7^ Department of Physics University of Guleph Guleph Ontario Canada; ^8^ Los Alamos National Laboratory Los Alamos NM USA

**Keywords:** Mars Science Laboratory Curiosity rover, chlorine, halite, salts, groundwater

## Abstract

The Mars Science Laboratory *Curiosity* rover is traversing a sequence of stratified sedimentary rocks in Gale crater that contain varied eolian, fluviodeltaic, and lake deposits, with phyllosilicates, iron oxides, and sulfate salts. Here, we report the chloride salt distribution along the rover traverse. Chlorine is detected at low levels (<3 wt.%) in soil and rock targets with multiple MSL instruments. Isolated fine‐scale observations of high chlorine (up to ≥15 wt.% Cl), detected using the ChemCam instrument, are associated with elevated Na_2_O and interpreted as halite grains or cements in bedrock. Halite is also interpreted at the margins of veins and in nodular, altered textures. We have not detected halite in obvious evaporitic layers. Instead, its scattered distribution indicates that chlorides emplaced earlier in particular members of the Murray formation were remobilized and reprecipitated by later groundwaters within Murray formation mudstones and in diagenetic veins and nodules.

## Key Points

- Isolated Cl enrichments in bedrock, in nodular textures, and at calcium sulfate vein margins, correlated with Na, indicate halite
- Mapping of Cl along the *Curiosity* traverse in Gale Crater indicates Cl enrichments are more common in select Murray formation members
- The scattered, isolated occurrences of chlorides are consistent with late groundwater reworking and remobilization of original deposits

## 1 Introduction

Evaporite mineral assemblages record the physical and chemical characteristics of past environments and allow us to place constraints on the chemistry of surface and subsurface fluids. In terrestrial environments, soluble chloride salts are typically among the last minerals to precipitate out of saline brines, preceded by various carbonates and sulfates, and are predicted to precipitate from fluids derived from basaltic weathering on Mars (Tosca & McLennan, 2006).

The Mars Odyssey Gamma Ray Spectrometer has mapped the global distribution of chlorine (Diez et al., 2009), and specific chloride‐enriched deposits were discovered in hundreds of irregular depressions in ancient terrains of the southern Martian highlands using the Mars Odyssey Thermal Emission Imaging System (Osterloo et al., 2010). These chlorides likely precipitated by evaporation from a ponded brine derived from groundwater upwelling and/or surface runoff. Chlorides can also form via efflorescence, the migration of saline fluids to the surface whereupon salts crystallize within sediment grains as thin crusts, as is thought to explain Cl‐enriched veneers and surface rinds at Meridiani Planum (Knoll et al., 2008) and Cl‐enriched soils and rock rinds at Gusev crater (Gellert et al., 2004; Haskin et al., 2005; Ming et al., 2006) detected by the Alpha Particle X‐ray Spectrometer (APXS) instruments on *Opportunity* and *Spirit*. Chlorine has been found in all Martian soils and dust at ~0.5–1 wt.% Cl (Berger et al., 2016; Cousin et al., 2017; Lasue et al., 2018; Yen et al., 2005). In situ soil studies have measured perchlorates (Hecht et al., 2009), and halite specifically has been detected in evaporitic mineral assemblages in the nakhlite meteorites (Bridges & Grady, 2000).

The Mars Science Laboratory *Curiosity* rover is investigating the stratigraphy of Mt. Sharp, the mound of sedimentary rocks filling the center of 155‐km Gale crater, which formed circa 3.8–3.6 Ga ago. Most of Gale's sedimentary rocks examined so far formed in a fluvio‐lacustrine environment, including both fluvial/alluvial deposits and laminated mudstones from subaqueous deposition (Grotzinger et al., 2015; Hurowitz et al., 2017; Rivera‐Hernández et al., 2019). Ca‐sulfates containing boron (Gasda et al., 2017), Mg‐sulfates (Rapin et al., 2019; submitted), desiccation features (Stein et al., 2018), and clay chemistries (Bristow et al., 2018) reported in Gale indicate past episodes of lake drying or lake level drop. Orbital surveys have not detected chlorides within Gale, but they are found in the nearby watershed of Sharp crater (Ehlmann & Buz, 2015). The Dynamic Albedo of Neutrons instrument is sensitive to Cl (Litvak et al., 2016), and APXS observations have found on average 1.0–1.4 wt.% Cl and up to 3.3 wt.% Cl in Gale's sedimentary rocks (O'Connell‐Cooper et al., 2017). Localized Cl enrichments have been reported in association with diagenetic raised ridges at Yellowknife Bay (Léveillé et al., 2014; McLennan et al., 2014), and halite has been reported in association with Ca‐sulfate veins (Forni et al., 2015; Nachon et al., 2014), but chlorine has not previously been systematically mapped in Gale crater sediments nor has a model for the origin, genesis, and distribution of these compounds been discussed.

In this paper we report the chlorine distribution in rocks and soils along Curiosity's traverse, using multiple instruments, in particular focusing on observations that indicate small‐scale enrichments in chloride salts, in order to inform our understanding of the depositional and groundwater environments at Gale crater. We report on their ChemCam detections as a function of stratigraphic level and target type and draw on supporting information from CheMin and SAM to identify the type of chloride salt present and determine its formation mechanism.

## 2 Methodology

APXS data from the arm‐mounted instrument, placed on or just above the surface of Mars, were used to determine bulk soil and rock Cl values over a spot size of 1.7–3 cm for 687 observations (up to Sol 2168), using the APXS standard calibration (Gellert et al., 2006).

ChemCam Laser‐Induced Breakdown Spectroscopy (LIBS) data from the mast‐mounted remote sensing instrument provided chemical analyses of >19,000 locations at fine scale (350‐ to 550‐μm diameter; Maurice et al., 2012) of targets typically 2–4 m away with Remote Micro‐Imager data for colocated context images (Maurice et al., 2012; Wiens et al., 2012). Major element compositions are calculated using multivariate techniques (R. Anderson, Clegg, et al., 2017; Clegg et al., 2017), but the detection and quantification of minor elements like Cl are complicated by relatively few, weak emission lines, interference with emission lines from other elements, and physical and chemical matrix effects. Neither APXS nor ChemCam can directly measure mineralogy and directly differentiate chlorides from perchlorates or chlorates, but they can infer mineralogy using correlations between elements.

Univariate analysis has been successfully applied to detect and quantify minor elements with ChemCam, for example, Li, Mn, and H (e.g., Lanza et al., 2014; Ollila et al., 2014; Payré et al., 2017; Rapin, Bousquet, et al., 2017; Thomas et al., 2018). We extended previous LIBS analyses of Cl in the laboratory (e.g., Anderson, Ehlmann, et al., 2017; Vogt et al., 2018) for analysis of ChemCam data. We apply standard ChemCam data preprocessing, removing the first five laser shots, which are contaminated by dust and subject to surface effects (as detailed in Wiens et al., 2013). We use the Cl emission line at 837.8 nm that increases monotonically with Cl content regardless of cation (Anderson, Ehlmann, et al., 2017) rather than the molecular emission from CaCl, which is complex and not easily used for direct quantification (Vogt et al., 2018). To quantify Cl, we fit the local region (831–841 nm) using methods described by Thomas et al. (2018) and report the fit Cl peak area. Before fitting, we normalize the shot‐averaged spectrum using the standard ChemCam Norm 3 method, which divides the spectra by the total detector intensity (in the case of Cl 838 nm, the VNIR detector—one of three in the instrument). While Rapin, Bousquet, et al. (2017) and Thomas et al. (2018) found normalization using C and O emission lines to work best in H quantification, Anderson, Ehlmann, et al. (2017) and additional lab measurement analyses done for this work indicate that Norm 3 provides the most linear calibrations with Cl concentration (see also section 4.2).

We constrained the ChemCam threshold of detection of Cl by three approaches. First, the threshold must be >0.03 wt.% Cl because no Cl peak is observed for the ChemCam calibration targets. The KGa‐2 calibration target contains 0.03 wt.% Cl, and the others have <0.01 wt.% Cl (Vaniman et al., 2012). Second, the threshold for loosely consolidated materials like soils must be less than ~1 wt.% Cl because a small Cl peak (peak area 0.83 × 10^‐4^) is seen in the dust, measured by the first shot of ChemCam analyses (Lasue et al., 2018). APXS measures 0.79–1.35 wt.% Cl in the Gale dust (Berger et al., 2016). Because of potential physical matrix effects, this same threshold may not apply to bedrock observations (e.g., Rapin, Meslin, et al., 2017; Thomas et al., 2018; and references therein). Third, the highest APXS Cl measurement in Gale is the bedrock target Stephen with 3.3 wt.% Cl, where ChemCam observes a small Cl peak (average Cl peak area 0.5 × 10^‐4^) indicating a threshold <3.3 wt.% Cl in rock. This performance on Mars is similar to laboratory studies that estimate a detection threshold of 3–6 wt.% Cl (Anderson, Ehlmann, et al., 2017) and >3 wt.% Cl (Gaft et al., 2014).

**Figure 1:**
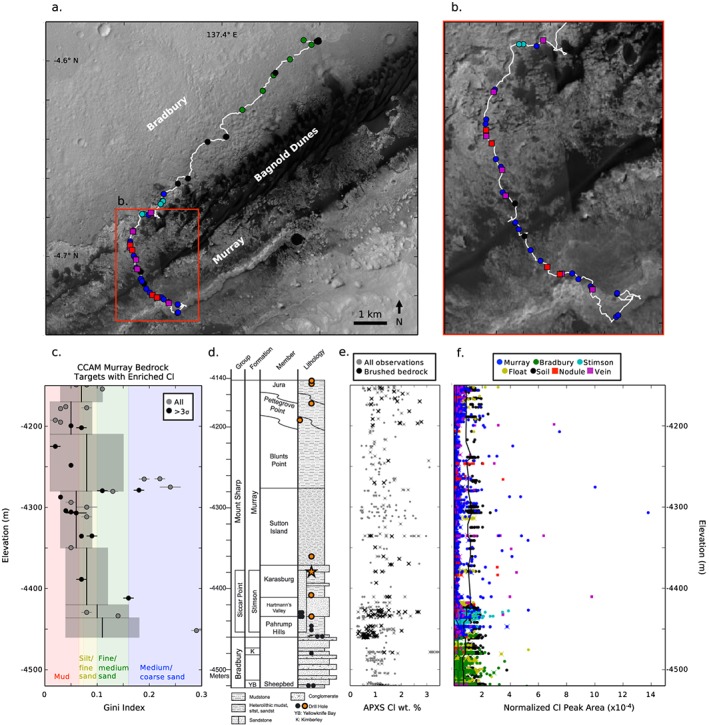
(a and b) Mars Science Laboratory traverse map showing the locations of ChemCam targets defined as Cl detections (Cl ≥ 2 × 10^‐4^ normalized peak area; or ≥3σ above the mean peak area) color coded by target type. (c) Gini index values for bedrock three‐sigma ChemCam (CCAM) Cl detections (black) and weaker Cl peaks (gray), which represent different grain size categories. Gini index mean (central line) and standard deviation (shaded bars) are reported for each member of Murray formation. (d) Gale crater stratigraphic column with drill sites, colored orange where no perchlorates are detected using Sample Analysis at Mars (Archer et al., 2019). CheMin detects halite at the Quela drill target (orange star; Achilles, 2018). (e) Alpha Particle X‐ray Spectrometer (APXS)‐measured Cl wt.% for all targets (gray) and brushed (relatively dust‐free) bedrock targets (black). (f) ChemCam normalized Cl peak area measurements. The lines show the moving average for the Murray bedrock (blue), Bradbury bedrock (green), and soil (black) points. Crosses indicate targets in common between APXS (e) and ChemCam (f).

Through mission Sol 2127, we examined all APXS data and all ChemCam spectra to identify targets containing Cl using the normalized Cl peak area. Then, using visual analysis of Remote Micro‐Imager and Mastcam images, we classified the targets as rock, soil, or diagenetic (veins or nodules) and localized them along the traverse and within Mt. Sharp geologic units (Figure 1). To estimate the grain size of ChemCam bedrock targets, we used the Gini index mean score, a composition‐based grain‐size proxy that uses point‐to‐point chemical variabilities in ChemCam data (Rivera‐Hernández et al., 2019), excluding points on or near diagenetic features.

## 3 Results

We observe Cl in all target types—soils, float rocks, bedrock, and diagenetic features (Figure 1). In soils the ChemCam Cl peak area varies little along the traverse, consistent with data from APXS showing ~1 wt.% Cl (Figure 1d; see also O'Connell‐Cooper et al., 2017). Most soils have normalized Cl peak areas of ~1–2 × 10^‐4^ (Figure 1e). Direct comparison of soil and rock Cl peak areas is complicated by physical matrix effects (e.g., Rapin, Meslin, et al., 2017; Thomas et al., 2018; and references therein), so it is unlikely they represent the same wt.% Cl, but characterizing relative variation is a useful benchmark. The Cl peak area moving average of soils is roughly constant with elevation along the traverse and three times higher than the average bedrock, regardless of formation. Most rock, vein, and nodule points do not have Cl peaks significantly above zero, indicating less than ~3 wt.% Cl. APXS results show brushed bedrocks on average have 0.4 wt.% more Cl than soils.

Bedrock ChemCam Cl peak areas show much greater variability than soils. Average Cl peak area is higher (50%) in the Bradbury and Stimson formations than the Murray formation (Figure 1e), but in the Murray we observe more high Cl peak areas, 3σ above the bedrock mean (≥2 × 10^‐4^). Stimson and Bradbury points have Cl peak areas up to 4 × 10^‐4^, whereas Murray points have values up to 14 × 10^‐4^. These very high Cl peak values occur in the Hartmann's Valley, Sutton Island, and Blunts Point members.

**Figure 2:**
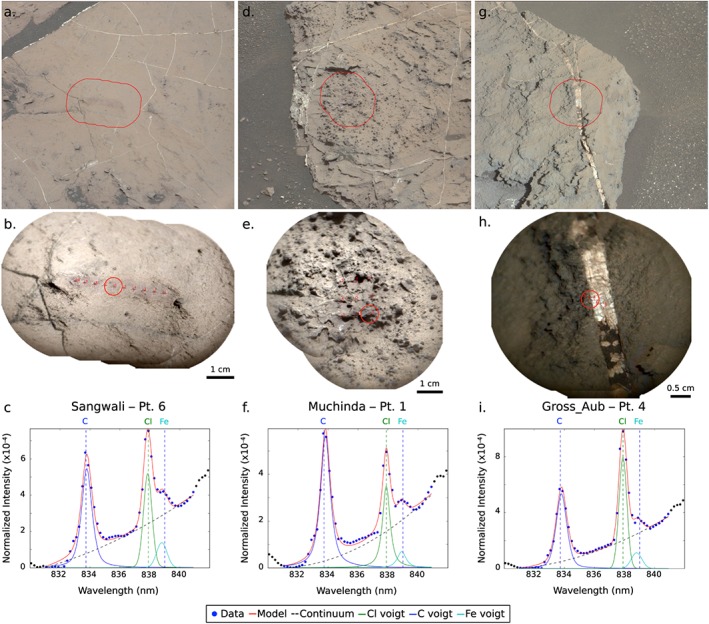
Mastcam (a, d, and g) and Remote Micro‐Imager images (b, e, and h) where Cl is detected including ChemCam spectra of the fit Cl peak at 838 nm (c, f, and i). Circles indicate the raster point where Cl is observed. Example targets shown include Sangwali, an isolated bedrock detection (a–c); Muchinda, a nodular detection (d–f); and Gross_Aub, a vein‐related detection (g–i). Mastcam images: mcam07482, mcam07156, and mcam05881.

The high ChemCam Cl observations (110 points) most frequently occur in isolated bedrock points (Figure 1e); that is, a single point within a raster (covering ~3 cm) contains a clear Cl peak (Figure 2c) but does not show textural or color differences compared to the nearby bedrock points without Cl (Figures 2a and 2b). Other detections are vein related, where Cl is most often detected at the edge of the Ca‐sulfate vein and the nearby bedrock (Figures 2g and 2h). In three cases Cl is seen in dark‐toned inclusions in veins (L'Haridon et al., 2018), and in one case Cl is seen within a vein (target Third_White_Ash). We have also detected Cl in targets with nodular, resistant textures (Figures 2d and 2e). For the majority of targets (~50%), the Cl peak in shot‐to‐shot profiles stays constant, but for 10 Murray points, the Cl peak increased to a maximum in the middle of the shot profile indicating that an isolated Cl‐rich grain or cement was measured.

Comparing the Murray bedrock targets containing high Cl with the Gini index mean score (Rivera‐Hernández et al., 2019) for each member of the Murray formation, we find that Cl detections occur more commonly in mudstones, siltstones, and fine sandstones relative to coarser grained rocks (Figure 1c). Cl detections in coarser sandstone occur at the Sutton Island and Blunts Point boundary. There are more rocks with high Cl higher stratigraphically, with units from the Sutton Point member onward having a greater number of high Cl points.

**Figure 3:**
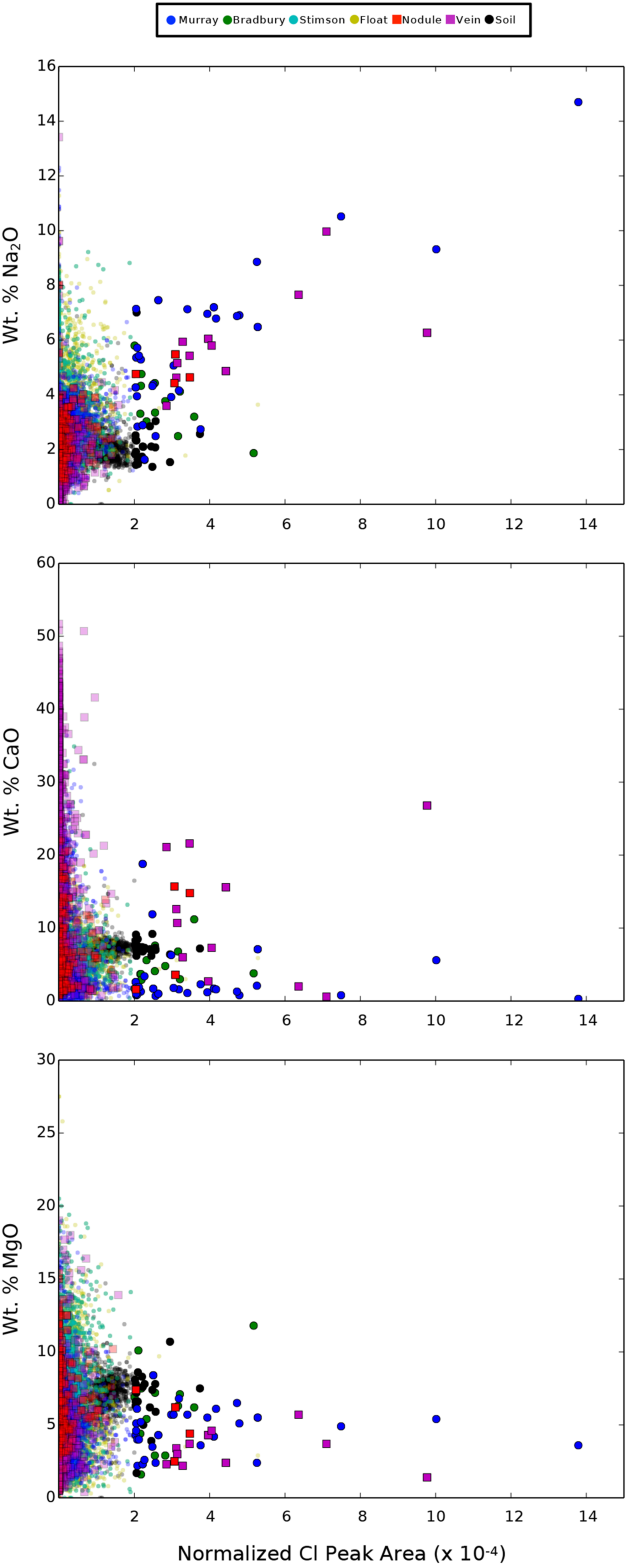
Normalized Cl peak area versus wt.% Na_2_O, CaO, and MgO from ChemCam. The opacity indicates the significance of the Cl observation. Fully opaque data points are three‐sigma Cl detections (≥ 2x10^‐4^ Cl peak area). No correlation is seen for CaO and MgO.

We observe a positive correlation between normalized Cl peak area and wt.% Na_2_O (Figure 3) and no apparent correlations between Cl peak area and wt.% CaO or MgO except for vein‐related targets where CaO enrichment is expected from mixing with the Ca‐sulfate vein (Figure 3). The Na wt.%‐Cl peak area correlation is most apparent for the Murray formation bedrock and vein‐related detections. The correlation suggests sodium chloride (NaCl), chlorate (NaClO_3_), or perchlorate (NaClO_4_) composition. We do not observe a correlation between O and Cl, so Na‐chlorate or Na‐perchlorate may be less likely, although LIBS data may not be very sensitive to variation in target O content (e.g., Schröder et al., 2019).

## 4 Discussion

### 4.1 Mineralogy

In bedrock, Cl peak areas are typically lower than the ChemCam detection threshold of ~3 wt.%, consistent with APXS brushed bedrock measurements showing on average 1.2 wt.% Cl (Figure 1d; O'Connell‐Cooper et al., 2017). ChemCam observes considerable Cl variation to higher values in the Murray formation bedrock (Figure 1e). We interpret the bedrock high Cl to be due to sporadic occurrences of chloride grains and/or cements within the bedrock. There is not an obvious correlation with texture or morphology in bedrock; detections are scattered.

The chloride is most likely NaCl, halite, based on the correlation between Cl and Na observed by ChemCam, and supporting data from CheMin and SAM. For the Quela drill target (star, Figure 1), where ChemCam measures a Cl peak in one point of the drill tailings (peak area 8 × 10^‐4^), CheMin reports 0.3 +/− 0.1 wt.% bulk halite (Achilles, 2018). The Sample Analysis at Mars Evolved Gas Analyzer measured O_2_ release below 600 °C has been interpreted as perchlorate (Sutter et al., 2017). Starting at the Oudam drill target (Sol 1364, elevation −4,435 m), Cl observed by APXS is no longer interpreted as perchlorate/chlorate because the <600 °C O_2_ release disappears (Figure 1c; Archer et al., 2019). Therefore, in the upper Murray, the Cl present measured by APXS is in the form of chlorides.

### 4.2 Quantification of Chlorine and Halite

ChemCam Cl peak area values have associated uncertainty from fitting the normalized spectra with an automated routine. The fit quadratic continuum sometimes cuts into the Cl peak, which could cause underestimation of the area. Additionally, a nearby minor Ti emission line (838.5 nm) that we do not fit could occasionally cause Cl peak area overestimation. Based on the outputs from the Levenberg‐Marquardt fit, the error in the fit Cl peak area, calculated by taking the square root of the diagonal elements of the covariance matrix, is <8%.

To constrain how much Cl high ChemCam peak areas represent, we applied the data processing methodologies described in section 2 to ChemCam lab model instrument measurements of Cl‐bearing samples (described in Anderson, Ehlmann, et al., 2017; Thomas et al., 2018). We tested normalization to the detector intensity and to C 248‐nm, C 834‐nm, and O 778‐nm peak areas. Due to differences in experimental conditions, translating the laboratory calibrations to Mars requires an Earth‐to‐Mars correction (Clegg et al., 2017). Because of large, wavelength‐dependent variability in the correction factor in the Cl wavelength region (831–841 nm), multiplying the lab data by this correction produced considerable variability in spectral shape and a more complicated continuum. Therefore, we multiplied the fit normalized Cl peak area by the average Earth‐to‐Mars correction in the Cl wavelength region. All normalizations were tested, and calibration curve fits were varied (linear and quadratic), resulting in a large range of 14.9–42.3 wt.% Cl for Point 4 of the bedrock target named aegis_post_1612a, which has the highest fit Cl peak area. Given qualitative examination of the spectra in comparison to laboratory mixtures of halite and basalt and the reported wt.% total of major oxides from partial least squares (81.3 wt.% total) for this observation point, high Cl values, that is, much greater than >20 wt.% Cl, are likely unrealistic. Future studies may refine the Cl quantification approach for Mars. As an additional constraint, assuming halite stoichiometry, using the ChemCam measured wt.% Na_2_O (14.7 +/− 1.5 wt.%), and subtracting an assumed Murray bedrock component (2.3–3.1 wt.% Na_2_O), we predict 13.8 +/− 2.2 wt.% Cl. This is on the lower end of the laboratory prediction. Thus, overall, the highest Cl point is estimated to result from ~15 wt.% Cl or ~25 wt.% halite, possibly with additional Cl associated with other phases.

### 4.3 Emplacement Models and Implications

Because our highest Cl observation corresponds to ~25 wt.% halite in bedrock, we are not observing pure halite at the LIBS scale of 350–550 μm. Instead, we are observing a mixture of bedrock and salt. For bedrock with chloride‐filled pores, ~25 wt.% chloride at ChemCam LIBS scale might be expected. Porosities of 20–40% are typical for fine‐grained sediments, though up to 80% porosity is possible for very fine, poorly consolidated mudstones (Fleury & Brosse, 2019). Because bedrock Cl detections are mostly in rocks with grain sizes less than the LIBS spot size, this implies either (1) there are large grains of halite (diameter greater than ~150 μm; larger than typical bedrock grainsize) that fill up greater than ~25 area% of the LIBS spot or (2) halite is a cement that in certain portions of the rock occupies all or part of the pore space.

The Sutton Island member of the Murray formation, where many potential chloride observations occur, is a package of heterolithic mudstones and sandstones likely deposited in lake and lake‐margin environments dominated by suspension fallout with less common traction deposits (Fedo et al., 2018). Bedrock enrichments of >30 wt.% Ca and Mg sulfates in Sutton Island and Blunts Point signal some of the beds may have formed in salty waters concentrated by evaporation (Rapin et al., 2019; submitted). Concretions and vertical and cross‐cutting Ca‐sulfate veins are common in the Sutton Island member and signify late diagenesis (Fedo et al., 2018; Rapin et al., 2019; submitted). We find Cl associated with high Na_2_O at the boundaries of some of the Ca‐sulfate veins observed in the Murray.

Fluids on Mars produced by basaltic weathering are typically Cl‐bearing and precipitate chloride salts during evaporation (Tosca & McLennan, 2006). In Gale crater, halite may have been emplaced initially as evaporitic salt layers, as mixed siliciclastic‐salt beds from evapo‐concentration of near‐surface waters, or during later diagenetic processes. Gasda et al. (2017) proposes that successive layers of chloride, sulfate, and borate salts were emplaced occasionally during the deposition of Mt. Sharp. Large‐scale, continuous beds of primary evaporite sequences have not been observed thus far by *Curiosity*, but sulfate layers remain to be explored (Milliken et al., 2010). As Mars transitioned to a drier climate, the Gale crater basin could have been analogous to a saline playa lake where acidic surface waters and alkaline groundwaters interacted to deposit clays and sulfates (Baldridge et al., 2009). Alternatively, Gale could represent a perennial lake system, which experienced multiple wet‐dry cycles where evaporite‐enriched deposits formed between mudstone deposits at the surface or in the shallow subsurface (Eugster & Hardie, 1978). Another alternative is that the chlorides precipitated from Cl‐rich brines during diagenetic processes with Cl derived from evaporation of fluids from thin layers now completely dissolved or layers yet‐to‐be encountered higher in the strata (e.g., Handford, 1991).

While it is difficult to determine the original halite emplacement mechanism with the available data, the occurrence of halite in particular fine‐grained members of the Murray suggests Cl‐rich brines were associated with these units specifically. Complementary lines of evidence such as desiccation features in the Murray (Stein et al., 2018), scattered thin beds enriched in sulfates (Rapin et al., 2019; submitted), as well as the heterolithic mudstones and sandstones observed in the Sutton Island member, indicate evaporation in a near‐shore environment may have been the initial halite source. The concentration of initial small‐scale primary deposits of chlorides to the Sutton Island and Blunts Point members of the Murray formations implies a transition in the Gale crater paleoenvironment and constrains later Gale lake waters to be episodically saline.

**Figure 4:**
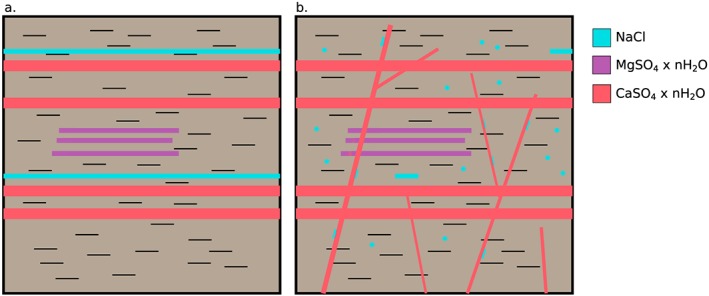
Potential emplacement scenario for chloride salts in the Murray formation. First, (a) halite (blue), Mg‐sulfates (purple), and Ca‐sulfates (red) enrichments form via evaporation of lake waters within siliclastics. Then, (b) sulfate‐bearing groundwaters precipitate additional Ca‐sulfates and mostly dissolve the halite, which reprecipitates as isolated grains or cements in the bedrock, in altered, nodular textures in the bedrock, and at the boundaries of Ca‐sulfate veins (red).

Halite is highly soluble and one of the easiest salts to later mobilize. We see a small number of halite detections most often as isolated enrichment points in bedrock targets, associated with Ca‐sulfate veins, or in nodular textures. Together, these observations are most consistent with reworking and remobilization by later groundwater (Figure 4). Following compaction and lithification of the Murray formation, late diagenetic fluids mobilized highly soluble salts like halite. The diagenetic fluids were likely SO_4_ rich, as they readily mobilized halite, mobilized Mg‐sulfate only to a limited degree (Rapin et al., 2019; submitted), and precipitated many Ca‐sulfate veins. These late‐stage fluids deposited Ca‐sulfates within fractures as well as chloride salts at vein margins. If the pressure from Ca‐sulfate precipitation forced fractures open as proposed by other analyses (Caswell & Milliken, 2017), halite would have precipitated last. Alternatively, the location on the edges of the fractures could also be consistent with a second fluid event after further fracturing between the bedrock and Ca‐sulfate vein. The nodular textures containing halite clearly represent diagenetic emplacement but the scattered, isolated bedrock detections are either remnants of where halite was emplaced initially or pore space where salts precipitated from later diagenetic fluids.

## 5 Conclusions

We present the first systematic study of chlorine and models for its emplacement in Gale crater using Mars Science Laboratory instruments. APXS measures Cl in bedrock and soils at 0.28–3.44 wt.% Cl. Cl is detected with the 838‐nm peak in ChemCam targets. Cl peaks are found in most soils. Most bedrock, vein, and nodule targets have no Cl at the ChemCam detection limit of ~3 wt.%, but sporadic occurrences of Cl are occasionally present in all these target types. For bedrock, the average Cl peak is higher in the Bradbury and Stimson formations than the Murray formation; however, the Murray contains isolated detections of high Cl (≥15 wt.% Cl). These correlate with high wt.% Na_2_O (~15 wt.%) and likely represent ~25 wt.% halite salt. CheMin detection of halite and Sample Analysis at Mars analyses, which indicate the presence of chlorides, corroborate halite. In addition to bedrock, halite is also detected in the Murray in nodular textures as well as at the outer boundaries of Ca‐sulfate veins. Halite bedrock detections occur in all stratigraphic intervals, but the highest values are in the Sutton Island, Blunts Point, Pettegrove Point, and Jura members. Given the solubility of halite and sporadic nature of its detection, we are likely observing halite emplaced by later groundwater reworking and remobilization of initial deposits. The restriction of high Cl to specific members of the Murray formation may indicate initial small‐scale primary deposits of chlorides, specific to these units, were locally remobilized by the fluids that precipitated Ca‐sulfates. Primary evaporitic chloride layers have not been observed thus far, but the concentration of deposits in particular members suggests an interval of more saline depositional waters and changes in the Gale crater paleoenvironment.
